# Detection of species and molecular typing of Leishmania in suspected patients by targeting cytochrome *b* gene in Zahedan, southeast of Iran

**DOI:** 10.14202/vetworld.2018.700-705

**Published:** 2018-05-26

**Authors:** Hadi Mirahmadi, Nasrin Rezaee, Ahmad Mehravaran, Peyman Heydarian, Saber Raeghi

**Affiliations:** 1 Infectious Diseases and Tropical Medicine Research Center, Zahedan University of Medical Sciences, Zahedan, Iran; 2 Department of Parasitology and Mycology, Faculty of Medicine, Zahedan University of Medical Sciences, Zahedan, Iran; 3 Department of Medical Parasitology and Mycology, School of Medicine, Qazvin University of Medical Sciences, Qazvin, Iran; 4 Department of Laboratory Sciences, Maragheh University of Medical Sciences, Maragheh, Iran

**Keywords:** cytochrome b, *Leishmania major*, *Leishmania tropica*, polymerase chain reaction-restriction fragment length polymorphism

## Abstract

**Aim:**

Cutaneous leishmaniasis (CL) is one of the most important health problems that are capable of involving both tropical and subtropical areas, especially in Iran. This cross-sectional study aimed to differentiate the species that are able to cause CL in Zahedan city by polymerase chain reaction-restriction fragment length polymorphism (PCR-RFLP) method.

**Materials and Methods:**

It was conducted on 145 suspected CL patients in Zahedan city between 2014 and 2016. The smears were initially prepared, air-dried, fixed with absolute methanol, and stained with 10% Giemsa. Then, we examined the stained samples by a light microscope under 1000× magnifications. PCR assay targeted cytochrome *b*(cyt *b*) gene using LCBF1 and LCBR2 primers and the products digested by Ssp1 enzymes.

**Results:**

From 145 suspected CL patients, 76 (52.4%) were positive in microscopic examination. In addition, we detected gene of interest (cyt *b*) in 98 (67.5%). The results of PCR-RFLP indicated that 53/98 (54%) cases were *Leishmania major* and 45/98 (46%) were *Leishmania tropica*, and the main species in these areas was *L. major*.

**Conclusion:**

We concluded that the microscopic examination is not sensitive enough and is not able to distinguish between different *Leishmania* species. Instead, molecular methods like PCR-RFLP can be appropriately used with promising results.

## Introduction

Leishmaniasis is a parasitic disease caused by various species of protozoa named *Leishmania* [1,2]. Cutaneous leishmaniasis (CL) is one of the 10 important parasitic diseases in tropical areas. The majority of CL cases in the Middle East have been reported from countries such as Afghanistan, Libya, Iraq, Iran, Jordan, Morocco, Saudi Arabia, Syria, Yemen, and Palestine [3]. Annual incidence of human leishmaniasis is estimated to be about 500,000, with 350 million people at the risk of this disease worldwide [4].

In Iran, CL is regarded as a serious health problem and is endemic in both urban and rural areas [5,6]. In addition, the annual new cases of CL in Iran are estimated to be 20,000 [7,8]. Zoonotic CL (ZCL) and anthroponotic CL (ACL) are both reported in Iran, mostly caused by *Leishmania major* and *Leishmania tropica*, respectively [9]. ZCL is endemic in various regions such as south-west and central of Iran with a high incidence rate and different reservoir hosts [10]. Due to differences in preventing and controlling methods used, clinical course, prognosis, and the method of treatment in applied against different species; the identification of species is of particular importance. Notably, *L. major* and *L. tropica* are morphological identical so that parasitological methods are not able to distinguish them from each other [11]. However, isoenzyme technique is a gold standard for differentiation of species, whereas molecular techniques are more expensive and time-consuming [12]. Polymerase chain reaction-restriction fragment length polymorphism (PCR-RFLP) has been suggested as an applicable molecular method for detecting and distinguishing different species of *Leishmania* [13-16]. Sistan and Baluchestan (a province in southeast Iran) are one of the most important endemic foci of disease [4,17].

Although there is a high prevalence of disease reported in these areas, identification of species has not yet been fully accomplished. Therefore, the present study attempted to differentiate the species of *Leishmania* that are capable of causing CL in Zahedan city by PCR-RFLP method.

This cross-sectional study aimed to differentiate the species that are able to cause CL in Zahedan city by PCR-RFLP method.

## Materials and Methods

### Ethical approval

This research was approved by the Faculty of Medicine, Zahedan University of Medical Sciences, Zahedan, Iran.

### Sampling and microscopic examination

This cross-sectional study was conducted on 145 suspected CL cases in Zahedan between 2014 and 2016. Data of cases were received from the health centers, and then their skin lesions were collected. These data consisted of the occupation, place of residence, type, number and location of lesions, were included in a questionnaire. Exudate smears were prepared, air-dried, fixed with absolute methanol, and stained with 10% Giemsa in a pH of 7.4 phosphate buffer for 20 min and examined by a light microscope under 1000× magnifications [18].

### DNA extraction

DNA was extracted from Giemsa stained slides with the High Pure PCR Template Purification (Takapouzist, Iran DynaBio Blood/Tissue Genomic DNA Extraction Kit) regarding the manufacturer’s procedure. The extracted DNA was stored at −20°C until the time of use.

### PCR assay

cyt *b* gene was amplified with a pair specific primers LCBF1 (5’GGTGTAGGTTTTAGTTT AGG3’) and LCBR2 (5’ CTACAATAAACAAAT CATAATATACAATT3’). The total volume of the reaction was 30 µl containing 3 µl DNA templates, 15.5 µl distilled water, 1 µl of each primer (forward and reverse), and 9.5 µl master mix (amplicon). PCR program used in the Thermocycler device (Eppendorf Mastercycler Gradient) was as follows: 95°C for 5 min (initial denaturation) followed by 35 cycles of these steps: 94°C for 45 s, 50°C for 1 min, 72°C for 1 min, and 72°C for 5 min.

Subsequently, 5 µL of PCR products was transferred into the 1.5% agarose gel (stained with ethidium bromide), and visualized by ultraviolet illumination. Finally, products with 880 bps length were considered as positive.

### PCR-RFLP

The Ssp1 enzymes were used to digest the PCR products in RFLP, 10 µl of PCR product, 1 µl of supplied restriction enzyme buffer, 1 µl of restriction enzyme diluted, and D.W up to 30 µl were provided for RFLP, according to enzyme directory. Subsequently, tubes were incubated at 37°C for 7 h. Digestion products were separated using 1.5% agarose gels in TAE buffer and visualized after staining by gel red.

### DNA sequencing and phylogenetic analysis

To confirm the diagnosis of species, PCR products were purified using PCR purification kit (Bioneer, Korea) and sequenced from both directions (Applied Biosystems, DNA Analyzers Sequencing, Bioneer, Korea, Sanger method), using the same primers as what used in the PCR. A neighbor-joining tree was gathered using the MEGA6 software. Results obtained and compared with the information presented in NCBI GenBank.

## Results

From 145 suspected CL patients (infected in different parts of Zahedan), 76 (52.4%) were positive in microscopic examinations, and 98 (67.5%) of samples were positive for cyt *b* gene in PCR. The distribution of patients according to diagnostic techniques is summarized in Table-1.

**Table-1:** The distribution of patients according to diagnostic technique.

Technique	Result	

	Positive (%)	Negative (%)
Microscopic	76 (52.41)	69 (47.59)
PCR	98 (67.58)	47 (32.42)

PCR: Polymerase chain reaction

**Figure-1:**
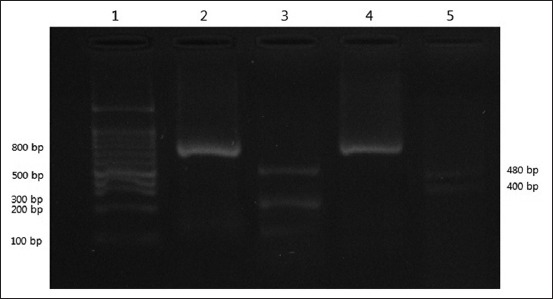
Restriction fragment length polymorphism patterns obtained from *Leishmania* stocks and Giemsa-stained smears. Lane 1. 100-bp size marker (fermentas); Lane 2. 4 Cyt *b*- polymerase chain reaction banding patterns (880 bp); Lane 3. *Leishmania tropica* (130 bp, 215 bp, 535 bp); Lane 4. *Leishmania major* (400 bp, 480 bp).

The results of PCR-RFLP indicated that 53/98 (54%) of cases were *L. major* and 45/98 (46%) were *L. tropica*. The main species in these areas were also shown to be *L. major*. Results are shown in Figure-1.

Of 53 samples of *L. major*, the most frequent lesions were on hands (26.4%), feet (20/4%), and face (15/1 %), respectively. While of 45 samples of *L. tropica*, the most frequent related lesions were on hands (28.9%), face (20%), and feet (15/6%), respectively (Table-2).

**Table-2:** The distribution of patients according to the location of lesions.

Type of parasite	Location of lesion n (%)												

	Face	Hand	Feet	Hand and face	Hand and trunk	Trunk	Hand and feet	Body	Head and face	Nose	Face and arms	Head and hand	Total
*L. major*	8 (15.1)	14 (26.4)	11 (20.7)	2 (3.8)	2 (3.8)	5 (9.4)	2 (3.8)	6 (11.3)	2 (3.8)	0 (0.0)	0 (0.0)	1 (1.9)	53 (100.0)
*L. tropica*	9 (20.0)	13 (28.9)	7 (15.7)	1 (2.2)	1 (2.2)	2 (4.4)	2 (4.4)	1 (2.2)	6 (13.4)	1 (2.2)	1 (2.2)	1 (2.2)	45 (100.0)
Negative	3 (6.4)	12 (25.5)	11 (23.4)	2 (4.3)	1 (2.1)	5 (10.6)	0 (0.0%)	3 (6.4)	0 (0.0)	0 (0.0)	10 (21.3)	0 (0.0)	47 (100.0)
Total	20 (13.8)	39 (26.9)	29 (20.0)	5 (3.4)	4 (2.8)	12 (8.3)	4 (2.8)	10 (6.9)	8 (5.5)	1 (0.7)	11 (7.5)	2 (1.4)	145 (100.0)

L. major=Leishmania major, L. tropica=Leishmania tropica

Another finding of this study was the number of lesions on each patient’s body. Of 53 cases with *L. major* had one lesion (49.1%) are the most frequent lesion then two and 18.9% had three lesions. Similarly, out of the 45 samples (51.1%) of the *L. tropica* showed one lesion on their body, while in 15.9% and 8.9% of cases there were two and three lesions, respectively. These results are shown in Table-3.

**Table-3:** Distribution of patients based on the number of lesions.

Type of parasite	Number of lesions										

	0*	1	2	3	4	5	6	9	10	12	Total
*L. major* n (%)	3 (5.7)	26 (49.1)	10 (18.9)	10 (18.9)	2 (3.8)	1 (1.9)	1 (1.9)	0 (0.0)	0 (0.0)	0 (0.0)	53 (100.0)
*L. tropica* n (%)	3 (6.7)	23 (51.1)	7 (15.6)	4 (8.9)	3 (6.7)	1 (2.2)	0 (0.0)	1 (2.2)	3 (6.7)	0 (0.0)	45 (100.0)
Negative n (%)	9 (19.1)	25 (53.2)	2 (4.3)	4 (8.5)	1 (2.1)	1 (2.1)	2 (4.3)	1 (2.1)	1 (2.1)	1 (2.1)	47 (100.0)
Total n (%)	15 (10.3)	74 (51.0)	19 (13.1)	18 (12.4)	6 (4.1)	3 (2.1)	3 (2.1)	2 (1.4)	4 (2.8)	1 (0.7)	145 (100.0)

* The purpose of this number was old wounds or wounds that were not active lesions and secretions and serosity were lacking, *L. tropica=Leishmania tropica, L. major=Leishmania major*

**Figure-2:**
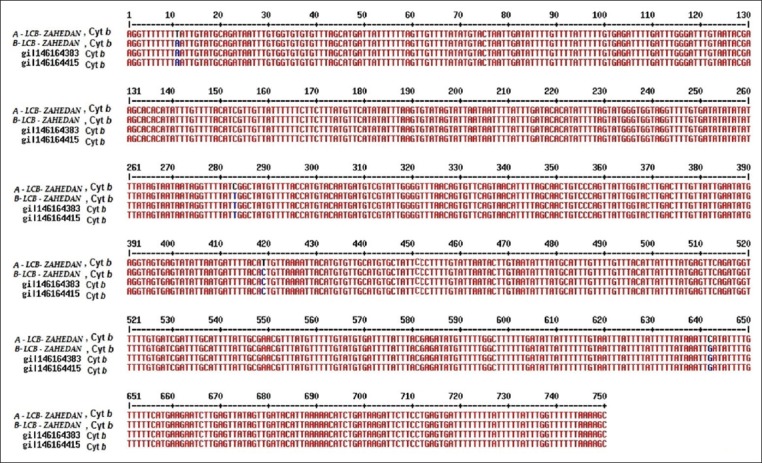
Comparison of cytochromes b gene sequences in *Leishmania major* isolates.

Results of cyt *b* sequencing of both *L. major* isolated from patients in Zahedan were compared with that of *L. major* isolates (cyt *b* gene) submitted in the GenBank using Multalin online software. These results are presented in Figure-2.

In the one isolate of *L. major*, there were five nucleotide changes between cyt *b*, predominantly in the third place of nucleotide codons (Wobble site) that did not lead to new amino acid creation.

**Figure-3:**
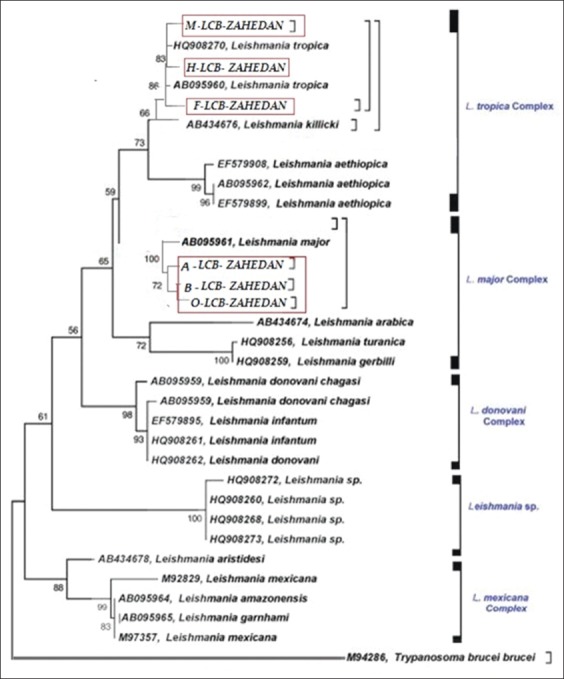
*Leishmania major* and *Leishmania tropica* (isolated from suspected cases in Zahedan) phylogenetic trees *Trypanosoma brucei* were considered as outside groups. The samples of this study are marked with different colored shapes. The rest of related to standard samples are prepared from the GenBank.

The isolates were then phylogenetically analyzed, based on the maximum likelihood method and using both the Tamura 2-parameter model sequences (obtained in this study) and the GenBank reference sequences (using the MEGA 6.0 Software) (Figure-3). A validation value 70% with a degree of confidence of 95% for topology in each branch was considered as acceptable. *Trypanosoma brucei* with access numbers cytochrome b: M94286 as the outside group was considered.

## Discussion

*Leishmania* is a parasitological disease with symptoms ranging from a mild cutaneous to a lethal visceral form. 12 million people in 88 countries are estimated to be suffering from this group of diseases [19]. CL is the most common form of disease worldwide. The CL is endemic in Iran, and about 20,000 new cases are reported annually from different parts of Iran [9].

Sistan and Baluchestan are one of the most important endemic foci of ACL and ZCL. Recently, there is an increasing rate of CL cases reported from this area [20]. This province has geographical importance because of the business, tourism, and exchanging visitors from neighboring countries. Zahedan is the old foci of CL in Iran. A vast majority of cases occur in this area due to the migration of people from villages across the border of Afghanistan and Pakistan [7,21]. In addition, for the reason of geographical feature of these villages and also being in the vicinity of residential areas with rodent’s nests, as well as the type of materials used in the construction of houses, barns, etc., they are regarded as ideal places for proliferation of sandflies and contribute to the distribution of disease. Due to the lack of a comprehensive study in these areas, the primary cause of the disease has been remained unclear since different procedures need to be used for preventing and controlling the CL, identification of the species is essential [22].

Sometimes, the traditional and microscopic examination methods used to detect species are not able to differentiate the species of parasites because of similarity in morphological features [23]. Isoenzyme is known as a gold standard method to distinguish between *Leishmania* species, but unfortunately, this method is expensive and time-consuming [24].

Molecular methods such as PCR-RFLP are valuable tools for differentiating of parasites that used in different microorganisms such as zoonotic worms and protozoa as *Leishmania* species and have been used in numerous studies [25-29].

There are various genetic markers suggested to be useful in molecular identification of *Leishmania* species including ITS, glycoprotein 63, mini-exon, kinetoplast DNA (kDNA), heat-shock proteins, cysteine protease B, cytochrome oxidase II, and the cyt *b* genes [30-32]. Cyt *b* gene, a fragment with high stability, is located in the mitochondrial genome of *Leishmania* spp. and has been widely used to identify genetic variations among the different species. In addition, this gene has shown to be beneficial in phylogenetic studies [33] and also is effective in phylogenetic studies [34]. This study applied PCR-RFLP method to define *Leishmania* species for not only arranging precise control platform precisely but also curing patients competently.

In the current study, from 145 CL-suspected cases in different parts of Zahedan City, 76 (52.4%) of them were positive in the microscopic examination, while in cytochrome b PCR 98 (67.5%) samples were positive. In a study conducted by Rasti, from 130 suspected CL patients, 87 (66.9%) cases were positive in the microscopic examination, and when using kDNA PCR 98 (75.4%) and nested PCR 96 (73.8%) cases were positive [35]. The results of PCR-RFLP indicated that 53/98 (54%) cases were *L. major* and 45/98 (46%) *L. tropica* and the main species in these areas was *L. major*, similar to the previous study performed in Mirjaveh, Sistan, and Baluchestan province [25]. In an attempt to identify the cutaneous and visceral leishmaniasis agents by molecular tests in specimens obtained from different geographical areas of Iran, the major cause of CL in Khuzestan, Ilam, Kermanshah, and Semnan (similarly to our study) was shown to be *L. major*, in Kurdistan province *L. tropica* and, in Tehran, North Khorasan, Esfahan, Kerman, Fars, and Razavi Khorasan; both species (*L. tropica* and *L. major*) of *Leishmania* were detected [2].

*L. major* as the main cause of CL in southern areas of Pakistan (bordering to Iran and Sistan and Baluchestan province) [36,37]. Besides, we showed that Sistan and Baluchestan are one of the most important foci of CL and resemble results presented in the previous study, *L. major* is the dominant cause of CL. In addition, to providing conventional control programs such as control the reservoir and vectors, it is crucial to monitor the migration of Pakistani immigrants and prevents the illegal traveling.

## Conclusion

The results of this study revealed that microscopic examination is not sensitive enough and is not a useful method in distinguishing different species of leishmaniasis. Instead, molecular approach such as PCR-RFLP can be widely used for this goal. Besides, our results showed that Sistan and Baluchestan are one of the important foci of CL and as were mentioned in a previous study in these areas, *L. major* is the dominant cause of CL. Moreover, to the using conventional control programs such as control the reservoir and vectors, it is crucial to monitor the migration of Pakistani immigrants and prevents of illegal traveling.

## Authors’ Contributions

HM assisted in the execution. NR attempted in the acquisition of data by laboratory techniques. SR and AM have designed, planned, and conducted this research. PH analyzed and interpreted the data. All authors have read and approved the final version of the manuscript.
